# Knowledge, Attitudes, and Hygienic Practices of Boiled Hypocotyls (*Borassus aethiopum* Mart) Vended in the Streets of Cotonou City and Its Outskirts, Benin

**DOI:** 10.1155/2018/4825435

**Published:** 2018-08-27

**Authors:** Brice M. Ohin, Kifouli Adéoti, Sonagnon S. Kouhoundé, Pacôme A. Noumavo, Sabine M. Ogoua, Nansirine Wabi, Marcellin C. Faïnou, Lamine Baba-Moussa, Fatiou Toukourou, Farid Baba-Moussa

**Affiliations:** ^1^Laboratoire de Microbiologie et des Technologies Alimentaires, Faculté des Sciences et Techniques, Université d'Abomey-Calavi, 04 BP 1107 Cotonou, Benin; ^2^Laboratoire de Biologie et de Typage Moléculaire en Microbiologie, Faculté des Sciences et Techniques, Université d'Abomey-Calavi, 05 BP 1604 Cotonou, Benin

## Abstract

In Benin, the boiled hypocotyl (*Borassus aethiopum* Mart) is one of the most common street foods consumed for its therapeutic properties. However, the knowledge, attitudes, and practices of boiled hypocotyl food sellers are poorly known despite the high contamination potential of these street foods. This study aimed to determine the knowledge, attitudes, and practices of boiled hypocotyls food vendors in the streets of Cotonou and its outskirts. The approach used concerned the process of good hygiene and production. Face-to-face interviews of 300 hypocotyls vendors and producers from Cotonou, Sèmè, Ouidah, and Abomey-Calavi were conducted. Concurrently, 200 samples of boiled hypocotyl were collected among study vendors. Results showed that all of the interviewed population (100%) of this activity are women. They are generally illiterate and 75% of them have never been to school. Additionally, 76.7% of street boiled hypocotyls sellers interviewed were mobile. The microbial profile of the boiled hypocotyls showed the lack of control and poor understanding of hygiene rules. The processes management also revealed two diagrams processes. The conditions in which hypocotyls vendors operate are largely unacceptable from a food safety point of view and an effort should be made to provide them with adequate infrastructure including potable water. In view of the socioeconomic impact of hypocotyl activities in Benin and their role in the unexpected arrival of food-borne diseases, vendors should be regularly trained in order to prevent outbreaks of food-borne illnesses.

## 1. Introduction

The* Borassus aethiopum* Mart., a nontimber forest product (NTFP) belonging to the family of* Arecaceae* is a multipurpose species found in the semiarid zones and subhumid tropical Africa, in the South of Asia, islands of the Pacific, and the Indian Ocean [1]. The* Borassus aethiopum *plant in communities is recognized and kept within the ecosystems for not only for its social importance but also for its economic role [2]. The different parts of* Borassus aethiopum *such as the wood, the roots, the petioles, the sheets, the final bud, the resin, the fruits, the seeds, and the sap are important sources of income for the rural communities especially for the women [2, 3]. Various modes of fruit consumption are known. The fruit can be directly boiled for eating or germinated before cooking. Indeed, the hypocotyl obtained after fruit germination is the best-selling derivative product consumed in the different streets of the cities of Benin. The hypocotyl is very appreciated by the rural and urban populations and Benin centre especially the department of Zou-Collines is and remains the centre of production and distribution.

According to Sokpon et al., approximately 90% of the households in Central Benin consume the products of the* Borassus aethiopum* including hypocotyls [4]. The high level of the consumption explains the interest of hypocotyls in the diet of the local population. Besides its use in the diet, the hypocotyl is also used for its medicinal properties. An ethnobotanic investigation revealed the aphrodisiac properties of hypocotyls in the treatment of erectile dysfunctions for the men [5]. Hypocotyls are consumed boiled or sometimes smoked [6]. Hypocotyl is largely vended in Benin city and, therefore, considered a street food.

Street foods are defined as ready-to-eat foods and beverages prepared and/or sold by vendors on the street from push-carts or buckets or balance poles or stalls or from shops having fewer than four permanent walls [7]. Street food represents the significant portion of the diet of many inhabitants in many major cities. The street vendors are conveniently situated, either in the living areas and or near the workplaces, and they provide a source of inexpensive, convenient, and comparatively nutritious food [8, 9]. A recent study [10] reported that the safety of street food was particularly affected by several factor as storage practice, the quality of the raw material, hygiene behaviours, and knowledge on microbiological quality. The boiled hypocotyls are widely sold in the streets of Cotonou and its outskirts.

However, the conditions of their processing and sanitary quality of the products are unknown.

Street foods are normally at risk of microbial contamination due to sales environment and failure to respect hygiene rules by vendors [11, 12]. Numerous studies showed that* Bacillus cereus, Salmonella *spp.,* Staphylococcus *spp.,* Escherichia coli, *and* Shigella *sp. and in other cases* Clostridum perfringers* [13–15] could be a potential source of microbial contamination of street vended foods.

Although there are many studies that have reported on the safety of street foods, there is no data on street vended boiled hypocotyl in cities. Therefore, there is need for understanding the hygiene knowledge and practices of hypocotyls vendors to ensure hygienic preparation of this street food. The objective of this work was to evaluate the knowledge, attitudes, and hygienic practices of vendors of boiled hypocotyls (*Borassus aethiopum* Mart) in Cotonou city.

## 2. Materials and Methods

### 2.1. Geographical Area

**Figure 1:**
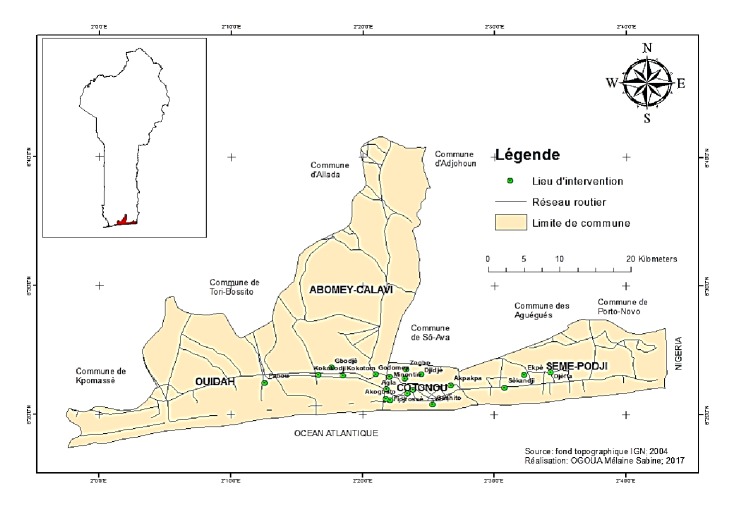
Map of Cotonou and its outskirts showing the study area.

The study was carried out in Cotonou city, the economic capital of Benin, and the surrounding districts of Southern Benin (Sèmè, Ouidah and Abomey-Calavi) (Figure 1). The districts were chosen due to their high population and concentration of hypocotyl street food vendors. The study was carried out during the period of August 2016 and May 2017. Data was collected at several points recognized for the sale and the purchase of hypocotyls. These included crossroads, markets, all streets, houses, and facades of churches.

### 2.2. Study Population

The population considered in this study comprised the producers and the vendors of boiled ready-to-eat hypocotyls of the* Borassus aethiopum* Mart. A total of 300 vendors and producers were selected and gave their consent to be enrolled, in which 180 come from various parts of the city of Cotonou, 50 from Sèmè district, 35 from Abomey-Calavi district, and 35 from Ouidah district. Before administering the questionnaire, a verbal explanation of the questionnaire and its content was given to the instructors of the selected population during programmed meetings, and their approval was obtained. In addition, total confidentiality was assured to the vendors and producers during the survey. After the interview step, 200 samples (140 in Cotonou, 24 in Sèmè, 20 in Abomey-Calavi and 16 in the Ouidah district) of boiled hypocotyl were collected for microbiological quality assessment. The sections of the questionnaire were developed below.

### 2.3. Questionnaire Design

A questionnaire that composed four sections was designed in order to assess the knowledge, attitudes, and hygienic practices of boiled hypocotyls vended in Cotonou and its peripheries. A set of questions previously used by [16] was modified and adapted to this study. Data was collected using observational checklist and a structured questionnaire by a face-to-face interview in the local dialect understood by the producers and sellers was conducted. These questions regarded sociodemographic characteristics of those responding (sex, age, instruction level, and kind of activity). Information relative to hypocotyl transformation conditions (the source of water; kind of packaging materials) was observed and recorded. Similarly, personal hygiene of sellers, environmental sanitation (point of sale, cleanliness of the selling site), and type of sellers (stationary or mobiles) were also indicated. Boiled hypocotyls production conditions were followed in order to note the diagram process. Particular attention was given to the working methods of the producers/sellers. Mishandling and unhygienic practices were recorded. After the interview step, the samples were collected to the vendors. Samples of the ready-to-eat hypocotyls were taken from vendors at the point of sale and hypocotyl supply. Each sample was placed in the sterile bag, closed immediately and kept in a cooler containing ice, and sent directly to the laboratory for the microbiological analyses.

### 2.4. Microbiological Analysis

Microbiological analysis was performed according to the standard methods. The buffer solution (peptone water containing 0.03g/L of tween 80) was used to revive the bacteria. Plate Count Agar (Biokar Diagnostics, France) was used to enumerate the total Mesophilic Aerobic Microorganisms on Petri dish after incubation at 30°C for 72 h. Yeasts and molds were enumerated on Sabouraud Agar containing Chloramphenicol (Sigma Aldrich, India) following incubation at 25°C for 5 days. VRBL Agar was used to evaluate the Total coliforms (TC) and Fecal Coliforms (FC) following incubation at 30°C/24 h and 44°C/48 h, respectively, for total coliform and fecal coliform, respectively. Tryptone Sulfite Neomycin Agar (Sigma Aldrich, Switzerland) was used for determination of anaerobic sulfite reducing (ASR) bacteria after incubation under strict anaerobic conditions. Baird Parker medium (Sigma Aldrich, Switzerland) medium supplemented with egg yolk and potassium tellurite was used to enumerate Staphylococcus flora following incubation at 37°C for 48 h. The lactic acid flora were enumerated on Man Rogosa Sharpe Agar (Sigma Aldrich, Switzerland) medium following incubation at 37°C for 48 h.

### 2.5. Data Management and Analysis

After manual tabulation and verification of the completion of the survey forms, data were encoded using the Excel 2010 and analyzed using SPSS software version 21. The results were summarized by descriptive statistics and restored in the form of switchboards of frequencies and the simple averages with the standard deviations or the reliable interval to 95%. In order to find significant correlations between different attributes and data, Pearson correlation coefficients were calculated. Statistical significance was set at the conventional cut-off of p < 0.05.

## 3. Results and Discussion

### 3.1. Sociodemographic Characteristics of Participants

Socioeconomic data including gender, age, educational level, and types of seller of the respondents is presented in Table 1. This survey revealed that all of the investigated, practicing study sample of this activity is constituted of women (100%, n = 300). They are all sellers of boiled hypocotyls (98.3%) and (1.7%) (n=51) and among them are producers of fresh hypocotyls. This study showed the gender profile of street food sellers and confirms the results found in others studies [16, 17].

The ages of the surveyed population varied from 15 to 50 years with mode of [18–23] which was the widest. Most of the sellers surveyed (43.3%) were between 30 to 40 years of age (Table 1). Other studies in the West Africa region have also reported that the average age of the street food vendor was 31-40 years [24].

The survey also revealed that women that sell hypocotyls are generally illiterate and 75% have never been to school. The rate of illiterate women is very high compared to other studies carried out on the street vendors in countries such as Uganda and Nigeria [10, 25]. This result should raise questions to the Beninese government with regard to the educational policies concerning the street food business.

Of all the street boiled hypocotyls vendors interviewed, only 23.3% maintained stationary post while the rest (76.7%) were mobile. These results are contrary to those reported by other researchers [26–28]. That found relatively a high proportion (between 70 and 92% [18, 24]) of stationary vendors in developing countries. The boiled hypocotyl is consumed at any time of the day as a snack in the same way like chips. Street boiled hypocotyl vendors are forced to move from street to street to sell their stock of hypocotyls. This requires a considerable physical effort and could be related to high level of young female involved in that activity.

### 3.2. Personal Hygiene Knowledge, Practices, and Process Conditions of Producers and Vendors: A Significant Correlation between Some Variables

Personal hygiene practices are extremely vital to ensure production of safe food to consumers [27]. The results of the survey showed that 90% (n = 270) of the study population had equipment reserved only for the transformation in the kitchen and for the sale. The hypocotyl was transformed in 41.7% (n = 124) of the outdoor cases. Within the framework of the transformation of the boiled hypocotyls, almost all of the vendors (98.3%) assert using detergents to wash their utensils. It was observed that 3.3% do not know that hand washing must be done before and after any tasks during the preparation of tubers. Previous work carried out in Vietnam showed that handwashing was a little-known concept to vendors on the streets [29].

However, it largely demonstrated that food handler's knowledge and attitudes are important factors that influence food safety and hygiene behaviours [30, 31]. In our study, although all study vendors (100%) reported having knowledge of hygiene practices, 5% (n=15) of them did not wash frequently kitchen utensils intended for the transformation. In addition, the majority (61.7%, n=185) of producers used tap water during the transformation similar to other studies [10, 16]. The vendors often used well water and, therefore, the containers used to keep the water could also be the source of the contamination [32] since this water is not treated at all. Likewise, this water could be used without treatment. In this study, among those that reported using well water, 23.3 (n = 70) % used it without treating it. The results revealed that the well water was treated or bleached during the processing. The method applied varied according to the processing location. There was a significant positive correlation (r = 0.307) that occurred between the water used for the process and the processing location (Table 4). On the other hand, the nature of the water used in the processing was significantly correlated with the instruction level of the vendors of hypocotyl. These facts showed a very narrow relation between the processing location, the packaging of the hot tubers and the used water which in turn, is correlated with the instruction level of the hypocotyls retailers.

**Figure 2:**
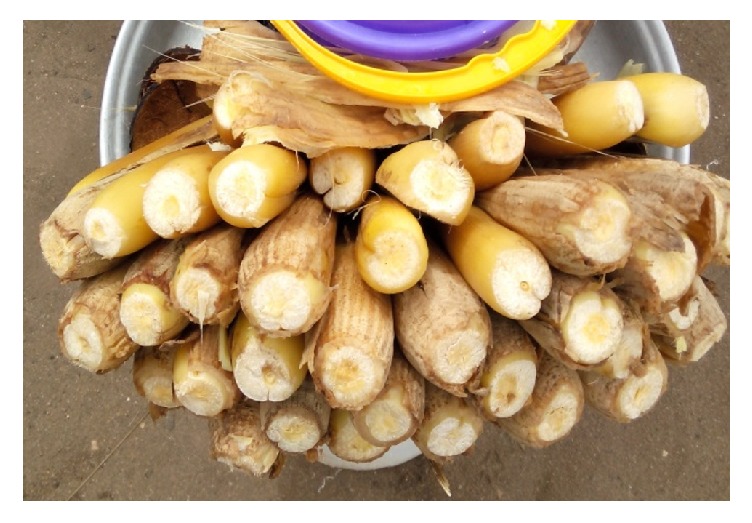
Photo of Boiled hypocotyl packaged in bulk and not covered by a drilled painting.

Although all the retailers of hypocotyls reported having knowledge of hygiene regulations, few applied them in reality. Several modes of hot packaging were utilized by the retailers of hypocotyls. Of these modes, the most commonly used method is the packaging in bulk and covering the boiled hypocotyls by perforated canvas (Figure 2). The second form of bulk (15%, 45 people) packaging was when hypocotyls are placed in a tray and left in the open (Table 2). A significant (r = 0.315) negative correlation was obtained between the water used for the process and the packaging of the hot tubers. Assays performed by [33] on the functional properties of the hypocotyl axes revealed that the water absorption capacity was 272.99 ± 15.13%. The packaging of the hypocotyls in bulk and covering them by a perforated canvas allowed aeration of the product. This aeration could facilitate the humidification of the product which could accelerate its alteration. On the other hand, this mode of packaging did not prevent contact of the product with the environment which is not free of microorganisms that can contaminate the product. Therefore, microorganisms are able to contaminate the hypocotyls and cause their alteration. This might be contributed to the cross-contamination [12, 34].

Analysis of food hygiene revealed that street foods have serious problems of cross-contamination [35]. It remains an important causative factor in outbreaks that occurred in street food.

Although all (100%, n = 300) the investigated vendors had knowledge of the existence of microorganisms and also knew that they can cause diseases, 86.7% had no idea of the ways of transmission of microorganisms (Table 3). What shows that hygiene regulations are not known and mastered by all and as well as there is a real lack of information on microorganisms and ways of contamination in a food. This explains the fact that very few retailers practice hygiene regulations and use the well water in the diagram process.

### 3.3. Heating as a Main Mode of Preservation of Unsold Boiled Hypocotyls

**Figure 3:**
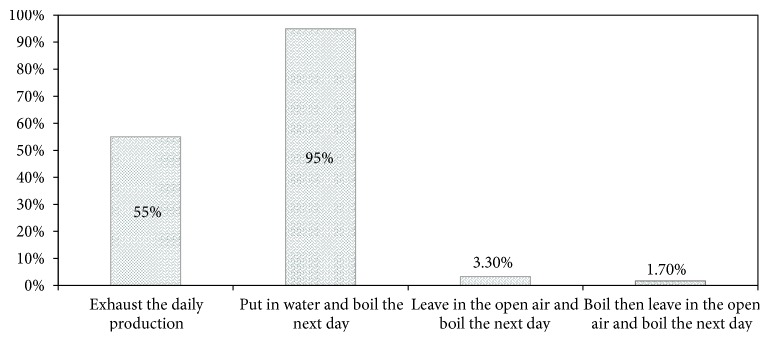
Preservation methods of unsold boiled hypocotyls used by vendors.

One of the most common problems in street foods is the fact that some sellers want to reprocess unsold foods to limit losses. Foods unsold on the day of preparation are usually treated and put out for display the next day. Reheating and refrigeration are the common treatment methods used to preserve unsold food [18, 19, 32]. This phenomenon induced* B. cereus cells* spores and* Staphylococci* multiplication when the foods were held on display [20]. In our study, with regard to unsold product, 55% of the women do their best to sell all their daily stocks of boiled hypocotyl because according to them, the next day tubers lose their organoleptic quality. To avoid losses, different methods were used by sellers to preserve leftover stock. Indeed, 95% of people studied left unsold boiled hypocotyls in the water until the next day and boiled it once again before selling, while 3.3% left them outdoors at night and boiled them the next day. It is noted that 1.7% of the boiled unsold hypocotyls the same day leave them outdoors until the next day and then boil them second time before reselling them (Figure 3).

Among the modes of preservation used by sellers, the most applied is the mode in which the hypocotyl is dipped into the water until the next day before being boiled again. This is the main mode of preservation of the boiled hypocotyls sold in the streets of Cotonou and its surroundings. Whatever the mode of preservation used, boiled hypocotyls remain exposed to microbial contamination. Indeed, fresh hypocotyls is rich with carbohydrates and proteins [6]. These elements make the hypocotyl a very rich environment favorable to the growth and development of microorganisms. Furthermore, if the water is contaminated by the pathogenic microorganisms, the degradation of these elements would be faster and, therefore, could modify the organoleptic quality of boiled hypocotyls. This could explain the loss of the organoleptic qualities of the tubers of hypocotyl dipped in water overnight.

### 3.4. Variability during Hypocotyl Processing

**Figure 4:**
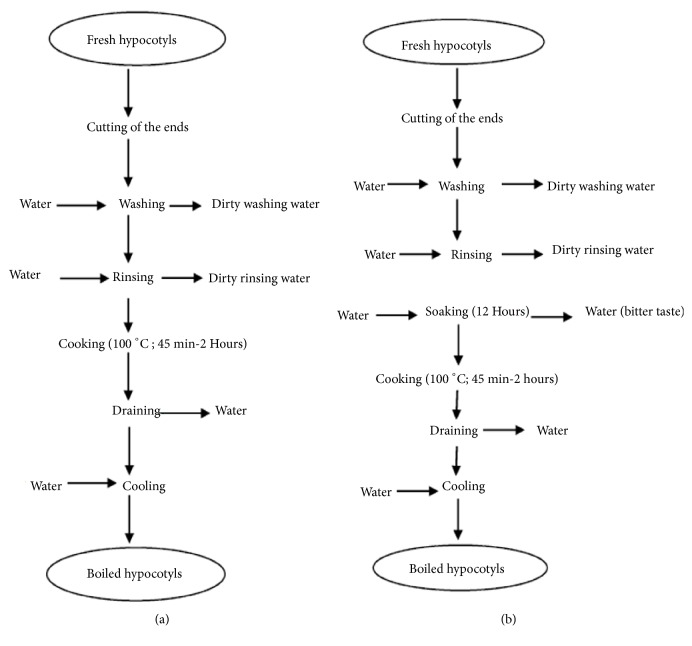
Process diagrams for preparation of boiled hypocotyls ((a) without soaking step and (b) with a soaking step). This soaking step in water during 12 hours could reduce antinutrients factor which is mainly present in the fresh hypocotyls and is responsible for its bitterness.

During the survey, two processes for boiled hypocotyls were identified (Figure 4) whereby process A is used by the majority (73.3%) of the retailers compared to Process B. According to the processes, Process A differs from the Process B by one operation: the soaking. Soaking entails dipping the hypocotyl into the water. According to those who use this practice, this technique decreases the bitterness of the hypocotyl. The stage of cooking lasted 45 min and could extend to two (2) hours at boiling temperature (90-100°C). This operation allowed cooking the hypocotyl and destroying microorganisms initially present on the hypocotyl. The water used for the cooking is either tap water or well water having undergone no heat treatment. The inadequate clean water and unhygienic working environments could be the cause of food poisoning cases [10, 27]. After cooking the product could be recontaminated by the water, utensils or working environment during draining, cooling, and packaging, [21, 22].

### 3.5. Microbiological Quality Profile: Prevalence of Pathogenic Bacteria in Boiled Hypocotyl

The microbiological quality profile of the boiled hypocotyls was evaluated by the enumeration of the aerobic flora total mesophile, lactic bacteria, total and fecal coliforms, Staphylococci, and anaerobic sulfite-reducers (ASR). Analyses revealed that the aerobic mesophile flora is abundant in the samples. The lactic bacteria, the total coliforms, and the fecal coliforms are found in all the samples of hypocotyl boiled. The ASR is the least found microorganisms. Only 14% of samples contain the ASR. Yeasts and molds are found in 88% of the analyzed samples and the Staphylococci in 90%. Molds were rarely found on samples contrary to yeasts.

Coliforms and Staphylococci are the most pathogenic bacteria predominant in the boiled hypocotyls. Major sources contributing to microbial contamination are the place of preparation, utensils for cooking and serving, raw materials, time and temperature abuse of cooked foods, and the personal hygiene of vendors [23, 36]. Various studies have identified the sources of food safety issues involved in street foods to be microorganism belonging to the genus* Bacillus, Staphylococcus, Clostridium, Vibrio, Campylobacter, Listeria, Salmonella, *and* E. coli* [11, 23, 32, 37]. In this work, results showed that samples are of unsatisfactory quality in 98% for the total mesophilic flora and 100% for the fecal coliforms. These results demonstrated the prevalence of the fecal coliforms in the boiled hypocotyls and identified this product as an important potential vehicle for food-borne diseases in Benin. The presence of fecal coliforms could be related to the use of untreated well water previously contaminated with animal and/or of bird's feces or by food handlers/vendors [38].

Hypocotyls sold in the streets of Cotonou and in the surroundings are of unsatisfactory microbiological quality based on our results. Generally, exceeding the criterion (“M” unacceptable quality) may reflect poor practices and alteration. This shows that hypocotyls are transformed into unsanitary conditions or are damaged. Indeed, during the process especially in hypocotyls cooking step (45 min to 2 hours) the microbial load initially present should significantly reduce. The germs found in the boiled hypocotyls therefore were probably associated with recontamination. This recontamination during the rest of the process could proceed from the processing environment, the conditioning, the water used in the processing, and the treatment of the remains or during the sale [10, 39]. The presence of the fecal coliforms in tubers indicates a fecal contamination of hypocotyls. These results show a real lack of hygiene during the transformation and during the hot packaging of hypocotyls. The abundance of these pathogens bacteria is due to the vendors having poor knowledge of food safety and nonapplication of the hygienic attitudes during the street food processing [29, 40]. This explains the loss of the organoleptic quality of the hypocotyls of the day before the processing. The microbiological and hygienic quality prove that very few retailers apply hygiene practice during hypocotyl process and vending.

## 4. Conclusion

The findings of this study clearly indicate that the knowledge and attitudes of most of the boiled hypocotyls vendors in the streets of Cotonou and its surroundings to food safety and hygiene were at low level. The processing and sale activities were carried out by mostly young and uneducated women. Processing and hot packaging conditions of hypocotyls indicate a lack of information and poor understanding of good hygiene practices. Totally, two diagram processes have been identified. The boiled hypocotyls have poor hygienic quality and have sources of coliforms and the Staphylococci. The results of this work could discourage consumers to eat street food in developing countries. Consumer's organizations could play key roles in food control system by calling attention to deficiencies. Particular attention could be paid to the transmission of fecal coliforms, Staphylococci and the water used during the process, the ustensils used in the sale, and the hygiene of the environment and hands. Ultimately, these measures would help to improve the street foods safety and preserve the consumers' health.

## Figures and Tables

**Table 1:** Social characteristics of the sellers of boiled hypocotyl.

**Variables **		**Frequencies**	**Percentage** **(n=300)**	**95**%** Confidence Interval **	
				**Lower bound**	**Upper bound**
Age	[15-20[	10	3.3	0.0	9.0
	[20-25[	45	15.0	7.0	25.4
	[25-30[	60	20.0	11.7	27.3
	[30-35[	80	26.7	14.6	40.4
	[35-40[	50	16.7	6.0	31.7
	[40-45[	35	11.7	1.7	17.3
	[45-50]	20	6.7	1.7	15.0
Educational level	Not literate	225	75.0	60.0	84.4
	Primary	70	23.3	13.7	36.3
	Secondary	05	1.7	0.0	5.7
Type of sellers	Stationary	70	23.3	13.3	38.7
	Mobiles	230	76.7	61.3	86.7

**Table 2:** Material and inputs used in the hypocotyl transformation and hygienic knowledge of vendor.

**Parameters**		**Frequency**	**Percentage**	**95**%** Confidence Interval**	
				**Lower bound**	**Upper bound**
Equipment reserved only for the transformation	No	30	10.0	3.3	17.3
	Yes	270	90.0	82.7	96.7

Place of transformation	Outdoors	125	41.7	26.7	52.3
	Kitchen	175	58.3	47.7	73.3

Washing with detergent	No	5	1.7	0.0	5.7
	Yes	295	98.3	94.3	100.0

Knowledge about handwashing	No	10	3.3	0.0	7.3
	Yes	290	96.7	92.7	100.0

Knowledge about hygiene	Yes	300	100.0	100.0	100.0

Water used for the transformation	Well water	115	38.3	24.3	48.3
	Tap water	185	61.7	51.7	75.7

Well water treatment	Adding javel	10	3.3	0.0	8.3
	Adding alum	35	11.7	4.3	19.0
	None	70	23.33	76.0	92.3

Frequency of cleaning of utensils	Rarely	15	5.0	0.0	11.3
	Often	285	95.0	88.7	100.0

Packaging of the hot tubers	In bulk and covered by a plastic bag	15	5.0	0.0	10.7
	Bulk and covered by a not drilled painting	20	6.7	1.0	14.7
	Others	45	15.0	6.7	24.0
	Bulk and covered by a drilled painting	220	73.3	63.7	84.0

**Table 3:** Knowledge of microbial contamination by the vendors of boiled hypocotyl in Cotonou and surrounding districts.

**Parameters**		**Frequencies**	**Percentage**	**95**%** Confidence Interval**	
				**Lower bound**	**Upper bound**
Knowledge of microorganisms	Yes	300	100.0	100.0	100.0

Knowledge of microorganisms as causes of the diseases	No	0	0	0	-
	Yes	300	100.0	100,0	100.0

Knowledge on the ways of transmission of microorganisms	No	-	-	-	-
	Unsuitable water	15	5.0	0.0	10.0
	Dirty hands	25	8.33	3.3	13.3
	No	260	86.7	79.3	94.0

**Table 4:** Bivariate correlation (Pearson Test) between survey variables.

Variable	City	Level of education	Processing location	Knowledge about handwashing	Water used for processing	Knowledge on the ways of transmission of microorganisms	Type of sellers	Packaging of the hot tubers	Treatment of leftovers
City	1								
Level of education	0.184	1							
Processing location	-0.186	-0.043	1						
Knowledge about handwashing	-0.051	0.106	-0.031	1					
Water used for processing	-0.207	-0.288^*∗*^	0.307^*∗*^	-0.045	1				
Knowledge on the ways of transmission of microorganisms	0.022	0.115	-0.160	0.071	-0.136	1			
Type of sellers	-0.101	-0,222	0.013	-0.117	-0.030	-0,211	1		
Packaging of the hot tubers	0.090	0.150	-0.343^*∗∗*^	-0.078	-0.315^*∗*^	-0.111	0.195	1	
Treatment of leftovers	-0.059	0.123	0.182	-0.040	0.059	-0.048	-0.136	-0.187	1

*∗*The correlation is significant at the 0.05 level (bilateral); *∗∗*the correlation is significant at the 0.01 level (bilateral).

## Data Availability

The data used to support the findings of this study are available from the corresponding author upon request.

## Supplementary Materials

Supplementary Materials The data used to support the findings of this study are included within the supplementary files. This section includes the survey sheet, the examination of survey cards, and the results of microbiological analyzes.
