# Supplementary material for: Knowledge, Attitudes, and Hygienic Practices of Boiled Hypocotyls (Borassus aethiopum Mart) Vended in the Streets of Cotonou City and Its Outskirts, Benin
Source: Biomed Res Int. 2018 Aug 27;2018:4825435. doi: 10.1155/2018/4825435 (PMC6129848; doi:10.1155/2018/4825435)
Supplement: Supplementary Materials — The data used to support the findings of this study are included within the supplementary files. This section includes the survey sheet, the examination of survey cards, and the results of microbiological analyzes. [file 4825435.f1.zip › 4825435.f1/Examination of survey cards.pdf]

Treatment of socio-economic data collected

**Questions**	**a**	**b**	**c**	**d**	**e**	**f**	**g**	**h**	**i**	**j**
Q1	300	0								
Q2	0	15		40	60	75	55	35	20	0
Q3	75	0	5	225						
Q4	0	300	0	0						
Q5	5	295								
Q6	295	5								
Q7	295	5								
Q8	270	30								
Q9	115	185	140	45						
Q10	295	5								
Q11	290	10								
Q12	300	0								
Q13	115	185	0							
Q14	10	30	0	255	5					
Q15	285	15								
Q16	300	0								
Q17	300	0								
Q18	15	35	20	230						
Q19	55	0								
Q20	0	300								
Q21	0	55								
Q22	55	245								
Q23	0	15	220	20	0	45				
Q24	165	220	20	5						
Q25	220	80								
Q1 : Sex	**100% are women**									
Q2 : Age	0%	5%	13,33%	20%	25%	18,33%	11,66%	6,66%	0%	
Q3: Level of education	25%	23,33%	1,66%	75%						
Q4: Kind of activity	1,66%	98,33%	0%	0%						
Q5: Production	1,66%	98,33%								
Q6: Procurement	98,33%	1,66%								
Q7 : Manufacturing	98,33%	1,66%								
Q8: Material	90%	10%								
Q9: Chicken	38,33%	61,66%	46,66%	15%						
Q10 : Washing	98,33%	1,66%								

Q11: Hands washing	96,66%	3,33%								
Q12:Hygiene rule	100%	0%								
Q13: Water used	38,33%	70%	0%							
Q14: Water treatment	3,33%	10%	0%	85%	1,66%					
Q15: Frequency of cleaning	95%	5%								
Q16 : Existence of microorganisms	100%	0%								
Q17: Microorganisms and deseases	100%	0%								
Q 18 : Pathways of transmission of microorganisms	5%	11,66%	6,66%	76,66%						
Q 19: Hygiene of the place of selling	18,33%	0%								
Q20: Hygiene of the vendor	0%	100%								
Q21: Presence of junk on the cooking site	0%	100%								
Q22: Seller type	18,33%	81,66%								
Q 23: Tubers packaging	0%	5%	73,33%	6,66%	0%	15%				
Q 24: Treatment of remains and conservation	55%	73%	3,33%	1,66%						
Q 25: Diagram	73,33%	26,66%								
	**Effective**	**Age**	**Percentage**			**Literate**			38,33%	70%
	0%	< 15	0			23,33%				
	500%	15-20	5			1,66%				
	13,33%	20-25	13,33	20		No literate				
	20%	25-30	20	25		75%				
	25%	30-35	25	30		Transmission pathways				
	18,33%	35-40	18,33	35	unsuitable water	5%				
	11,66%	40-45	11,66	40						
	6,66%	45-50	6,66	45	Dirty hands	11,66%				
	0%	>50	0	50						
					Dirty utensils	6,66%				

					Unknown	76,66%				
**Tuber packaging**										
Covered by a plastic bag	5%									
Covered by a non-perforated canvas	73,33%									
Covered by a perforated canvas	6,66%									
Other (not covered)	15%									
**Unsold processing and conservation**										
Exhaust daily production	55%									
Put in water and boil the next day	95%									
Leave in the open air and boil the next day	3,30%									
Boil, put the air out and boil again the next day	1,70%									
**Water used**										
Well water	38,33%									
Tap water	70%									
Untreated well water	60,86%									
Treated well water	39,13%									
