# Supplementary material for: Knowledge, Attitudes, and Hygienic Practices of Boiled Hypocotyls (Borassus aethiopum Mart) Vended in the Streets of Cotonou City and Its Outskirts, Benin
Source: Biomed Res Int. 2018 Aug 27;2018:4825435. doi: 10.1155/2018/4825435 (PMC6129848; doi:10.1155/2018/4825435)
Supplement: Supplementary Materials — The data used to support the findings of this study are included within the supplementary files. This section includes the survey sheet, the examination of survey cards, and the results of microbiological analyzes. [file 4825435.f1.zip › 4825435.f1/Results of Microbiology Analyzes.pdf]

Samples	Aerobic total mesophilic flora	Yeast and mould	Staphylo-coccus	Total Coliforms	Fecal Coliforms	Lacto-bacillus	Sulphito-reductive anaerobes
	CFU/g						
S1	1913636,36	650	12727,27	149090,91	141818,18	1577272,7	70
S2	9090909,09	500	60090,91	1800000	1672727,3	187727,27	10
S3	13663636,36	450	120909,09	2559090,9	1481818,2	3972727,3	0
S4	1359090,91	50	2181,82	257272,73	31000	315454,55	0
S5	1709090,91	2300	3000	355909,09	231363,64	365909,09	55
S6	2113636364	550	14045,45	23272727	2259090,9	1,477E+09	0
S7	913636,36	386,36	545,45	954,55	450	768181,82	0
S8	936363636,4	6090,91	23863,64	273181,82	120909,09	904545455	0
S9	237727,27	2031,82	281,82	169545,45	81818,18	111363,64	0
S10	1622727,27	0	350	107272,73	72727,27	1177272,7	0
S11	1363636,36	1045,45	200	123636,36	99545,45	1150000	0
S12	265454,55	0	0	56818,18	20000	187272,73	0
S13	17909090,91	550	1300	3313636,4	1304545,5	2018181,8	0
S14	1800000	150	950	1659090,9	204545,45	272727,27	10
S15	1845454,55	818,18	6227,27	1318181,8	281818,18	325454,55	0
S16	3559090,91	1045,45	125000	1186363,6	886363,64	2327272,7	0
S17	1536363,64	100	61636,36	454545,45	204545,45	293636,36	0
S18	3445454,55	100	1136,36	3018181,8	1804545,5	239090,91	20
S19	1572727,27	1272,73	1600	183181,82	105909,09	267727,27	5
S20	1272727,27	2000	1700	112727,27	86818,18	278636,36	0
S21	2200000	200	11818,18	326363,64	106818,18	1095454,6	0
S22	895454,55	809,09	0	125909,09	33863,64	272727,27	0
S23	190909,09	190909,09	200	85454,55	33181,82	30000	0
S24	331818,18	2318,18	1363,64	227272,73	95454,55	86363,64	0
S25	514259,48	70,71	0	1359090,9	731818,18	2454545,5	35
S26	8681818,18	227,27	5181,82	359090,91	240909,09	5954545,5	0
S27	18590909,09	50	668,18	12181818	9636363,6	2009090,9	0
S28	477272,73	750	250	286363,64	109090,91	24545,45	0
S29	995454,55	200	804,55	131818,18	92727,27	572727,27	0
S30	9136363,64	200	863,64	65909,09	20000	8409090,9	0
S31	1236363,64	100	1000	59545,45	45909,09	936363,64	0
S32	2445454,55	0	140,91	136818,18	109090,91	1936363,6	0
S33	945454,55	0	1363,64	56363,64	29545,45	659090,91	0
S34	40909,09	572,73	568,18	9954,55	1500	18181,82	0
S35	277272,73	0	0	19090,91	8045,45	195454,55	0
S36	1309090,91	377,27	1136,36	144090,91	95909,09	963636,36	0
S37	1313636,36	590,91	1863,64	110909,09	87272,73	981818,18	0
S38	1272727,27	1272,73	909,09	142727,27	87272,73	236363,64	0
S39	1472727,27	1636,36	2045,45	265909,09	220000	269545,45	0
S40	1159090,91	286,36	7727,27	422727,27	180909,09	257272,73	0
S41	118181,82	472,73	768,18	11818,18	6818,18	213636,36	0
S42	500000	0	0	12727,27	5318,18	18181,82	0
S43	8454545,46	954,55	1190,91	3018181,8	1786363,6	1886363,6	0
S44	90454545,45	3454,55	18136,36	668181,82	200000	89545455	0
S45	918181,82	650	2350	95000	59090,91	299090,91	0
S46	1118181,82	150	200	130909,09	88181,82	690909,09	0
S47	1963636,36	2000	1772,73	128181,82	90000	1222727,3	0
S48	1009090,91	1045,45	550	70000	47727,27	936363,64	0
S49	281818,18	227,27	1000	11045,45	5909,09	227272,73	0
S50	1286363,64	1045,45	954,55	99545,45	79090,91	1077272,7	0
S51	9136363,64	200	863,64	65909,09	20000	8409090,9	0
S52	1236363,64	100	1000	59545,45	45909,09	936363,64	0
S53	2445454,55	0	140,91	136818,18	109090,91	1936363,6	0
S54	945454,55	0	1363,64	56363,64	29545,45	659090,91	0

S55	40909,09	572,73	568,18	9954,55	1500	18181,82	0
S56	277272,73	0	0	19090,91	8045,45	195454,55	0
S57	1309090,91	377,27	1136,36	144090,91	95909,09	963636,36	0
S58	1313636,36	590,91	1863,64	110909,09	87272,73	981818,18	0
S59	1272727,27	1272,73	909,09	142727,27	87272,73	236363,64	0
S60	1472727,27	1636,36	2045,45	265909,09	220000	269545,45	0
S61	1159090,91	286,36	7727,27	422727,27	180909,09	257272,73	0
S62	1118181,82	150	200	130909,09	88181,82	690909,09	0
S63	1963636,36	2000	1772,73	128181,82	90000	1222727,3	0
S64	1009090,91	1045,45	550	70000	47727,27	936363,64	0
S65	281818,18	227,27	1000	11045,45	5909,09	227272,73	0
S66	1286363,64	1045,45	954,55	99545,45	79090,91	1077272,7	0
S67	1536363,64	100	61636,36	454545,45	204545,45	293636,36	0
S68	3445454,55	100	1136,36	3018181,8	1804545,5	239090,91	20
S69	1572727,27	1272,73	1600	183181,82	105909,09	267727,27	5
S70	1272727,27	2000	1700	112727,27	86818,18	278636,36	0
S71	2200000	200	11818,18	326363,64	106818,18	1095454,6	0
S72	895454,55	809,09	0	125909,09	33863,64	272727,27	0
S73	190909,09	190909,09	200	85454,55	33181,82	30000	0
S74	331818,18	2318,18	1363,64	227272,73	95454,55	86363,64	0
S75	514259,48	70,71	0	1359090,9	731818,18	2454545,5	35
S76	8681818,18	227,27	5181,82	359090,91	240909,09	5954545,5	0
S77	18590909,09	50	668,18	12181818	9636363,6	2009090,9	0
S78	477272,73	750	250	286363,64	109090,91	24545,45	0
S79	995454,55	200	804,55	131818,18	92727,27	572727,27	0
S80	118181,82	472,73	768,18	11818,18	6818,18	213636,36	0
S81	500000	0	0	12727,27	5318,18	18181,82	0
S82	8454545,46	954,55	1190,91	3018181,8	1786363,6	1886363,6	0
S83	90454545,45	3454,55	18136,36	668181,82	200000	89545455	0
S84	918181,82	650	2350	95000	59090,91	299090,91	0
S85	17909090,91	550	1300	3313636,4	1304545,5	2018181,8	0
S86	1800000	150	950	1659090,9	204545,45	272727,27	10
S87	1845454,55	818,18	6227,27	1318181,8	281818,18	325454,55	0
S88	3559090,91	1045,45	125000	1186363,6	886363,64	2327272,7	0
S89	1536363,64	100	61636,36	454545,45	204545,45	293636,36	0
S90	3445454,55	100	1136,36	3018181,8	1804545,5	239090,91	20
S91	1572727,27	1272,73	1600	183181,82	105909,09	267727,27	5
S92	1272727,27	2000	1700	112727,27	86818,18	278636,36	0
S93	2200000	200	11818,18	326363,64	106818,18	1095454,6	0
S94	895454,55	809,09	0	125909,09	33863,64	272727,27	0
S95	190909,09	190909,09	200	85454,55	33181,82	30000	0
S96	331818,18	2318,18	1363,64	227272,73	95454,55	86363,64	0
S97	514259,48	70,71	0	1359090,9	731818,18	2454545,5	35
S98	1622727,27	0	350	107272,73	72727,27	1177272,7	0
S99	1363636,36	1045,45	200	123636,36	99545,45	1150000	0
S100	265454,55	0	0	56818,18	20000	187272,73	0
S101	17909090,91	550	1300	3313636,4	1304545,5	2018181,8	0
S102	1800000	150	950	1659090,9	204545,45	272727,27	10
S103	1845454,55	818,18	6227,27	1318181,8	281818,18	325454,55	0
S104	3559090,91	1045,45	125000	1186363,6	886363,64	2327272,7	0
S105	1913636,36	650	12727,27	149090,91	141818,18	1577272,7	70
S106	9090909,09	500	60090,91	1800000	1672727,3	187727,27	10
S107	13663636,36	450	120909,09	2559090,9	1481818,2	3972727,3	0
S108	1359090,91	50	2181,82	257272,73	31000	315454,55	0
S109	1709090,91	2300	3000	355909,09	231363,64	365909,09	55
S110	2113636364	550	14045,45	23272727	2259090,9	1,477E+09	0
S111	913636,36	386,36	545,45	954,55	450	768181,82	0
S112	936363636,4	6090,91	23863,64	273181,82	120909,09	904545455	0
S113	237727,27	2031,82	281,82	169545,45	81818,18	111363,64	0

S114	1622727,27	0	350	107272,73	72727,27	1177272,7	0
S115	1913636,36	650	12727,27	149090,91	141818,18	1577272,7	70
S116	9090909,09	500	60090,91	1800000	1672727,3	187727,27	10
S117	13663636,36	450	120909,09	2559090,9	1481818,2	3972727,3	0
S118	1359090,91	50	2181,82	257272,73	31000	315454,55	0
S119	1709090,91	2300	3000	355909,09	231363,64	365909,09	55
S120	2113636364	550	14045,45	23272727	2259090,9	1,477E+09	0
S121	913636,36	386,36	545,45	954,55	450	768181,82	0
S122	936363636,4	6090,91	23863,64	273181,82	120909,09	904545455	0
S123	237727,27	2031,82	281,82	169545,45	81818,18	111363,64	0
S124	2445454,55	0	140,91	136818,18	109090,91	1936363,6	0
S125	945454,55	0	1363,64	56363,64	29545,45	659090,91	0
S126	1622727,27	0	350	107272,73	72727,27	1177272,7	0
S127	1363636,36	1045,45	200	123636,36	99545,45	1150000	0
S128	265454,55	0	0	56818,18	20000	187272,73	0
S129	17909090,91	550	1300	3313636,4	1304545,5	2018181,8	0
S130	1800000	150	950	1659090,9	204545,45	272727,27	10
S131	1845454,55	818,18	6227,27	1318181,8	281818,18	325454,55	0
S132	3559090,91	1045,45	125000	1186363,6	886363,64	2327272,7	0
S133	1536363,64	100	61636,36	454545,45	204545,45	293636,36	0
S134	3445454,55	100	1136,36	3018181,8	1804545,5	239090,91	20
S135	1572727,27	1272,73	1600	183181,82	105909,09	267727,27	5
S136	1272727,27	2000	1700	112727,27	86818,18	278636,36	0
S137	2200000	200	11818,18	326363,64	106818,18	1095454,6	0
S138	895454,55	809,09	0	125909,09	33863,64	272727,27	0
S139	190909,09	190909,09	200	85454,55	33181,82	30000	0
S140	331818,18	2318,18	1363,64	227272,73	95454,55	86363,64	0
S141	514259,48	70,71	0	1359090,9	731818,18	2454545,5	35
S142	8681818,18	227,27	5181,82	359090,91	240909,09	5954545,5	0
S143	18590909,09	50	668,18	12181818	9636363,6	2009090,9	0
S144	477272,73	750	250	286363,64	109090,91	24545,45	0
S145	995454,55	200	804,55	131818,18	92727,27	572727,27	0
S146	9136363,64	200	863,64	65909,09	20000	8409090,9	0
S147	1236363,64	100	1000	59545,45	45909,09	936363,64	0
S148	2445454,55	0	140,91	136818,18	109090,91	1936363,6	0
S149	945454,55	0	1363,64	56363,64	29545,45	659090,91	0
S150	40909,09	572,73	568,18	9954,55	1500	18181,82	0
S151	277272,73	0	0	19090,91	8045,45	195454,55	0
S152	1309090,91	377,27	1136,36	144090,91	95909,09	963636,36	0
S153	1313636,36	590,91	1863,64	110909,09	87272,73	981818,18	0
S154	1272727,27	1272,73	909,09	142727,27	87272,73	236363,64	0
S155	1472727,27	1636,36	2045,45	265909,09	220000	269545,45	0
S156	1159090,91	286,36	7727,27	422727,27	180909,09	257272,73	0
S157	118181,82	472,73	768,18	11818,18	6818,18	213636,36	0
S158	500000	0	0	12727,27	5318,18	18181,82	0
S159	8454545,46	954,55	1190,91	3018181,8	1786363,6	1886363,6	0
S160	90454545,45	3454,55	18136,36	668181,82	200000	89545455	0
S161	918181,82	650	2350	95000	59090,91	299090,91	0
S162	1118181,82	150	200	130909,09	88181,82	690909,09	0
S163	1963636,36	2000	1772,73	128181,82	90000	1222727,3	0
S164	40909,09	572,73	568,18	9954,55	1500	18181,82	0
S165	277272,73	0	0	19090,91	8045,45	195454,55	0
S166	1309090,91	377,27	1136,36	144090,91	95909,09	963636,36	0
S167	1313636,36	590,91	1863,64	110909,09	87272,73	981818,18	0
S168	913636,36	386,36	545,45	954,55	450	768181,82	0
S169	936363636,4	6090,91	23863,64	273181,82	120909,09	904545455	0
S170	237727,27	2031,82	281,82	169545,45	81818,18	111363,64	0
S171	1009090,91	1045,45	550	70000	47727,27	936363,64	0
S172	281818,18	227,27	1000	11045,45	5909,09	227272,73	0

S173	1286363,64	1045,45	954,55	99545,45	79090,91	1077272,7	0
S174	1272727,27	1272,73	909,09	142727,27	87272,73	236363,64	0
S175	1472727,27	1636,36	2045,45	265909,09	220000	269545,45	0
S176	1159090,91	286,36	7727,27	422727,27	180909,09	257272,73	0
S177	118181,82	472,73	768,18	11818,18	6818,18	213636,36	0
S178	500000	0	0	12727,27	5318,18	18181,82	0
S179	8454545,46	954,55	1190,91	3018181,8	1786363,6	1886363,6	0
S180	90454545,45	3454,55	18136,36	668181,82	200000	89545455	0
S181	477272,73	750	250	286363,64	109090,91	24545,45	0
S182	995454,55	200	804,55	131818,18	92727,27	572727,27	0
S183	9136363,64	200	863,64	65909,09	20000	8409090,9	0
S184	1236363,64	100	1000	59545,45	45909,09	936363,64	0
S185	1363636,36	1045,45	200	123636,36	99545,45	1150000	0
S186	265454,55	0	0	56818,18	20000	187272,73	0
S187	8681818,18	227,27	5181,82	359090,91	240909,09	5954545,5	0
S188	18590909,09	50	668,18	12181818	9636363,6	2009090,9	0
S189	918181,82	650	2350	95000	59090,91	299090,91	0
S190	1118181,82	150	200	130909,09	88181,82	690909,09	0
S191	1963636,36	2000	1772,73	128181,82	90000	1222727,3	0
S192	1009090,91	1045,45	550	70000	47727,27	936363,64	0
S193	281818,18	227,27	1000	11045,45	5909,09	227272,73	0
S194	1286363,64	1045,45	954,55	99545,45	79090,91	1077272,7	0
S195	1913636,36	650	12727,27	149090,91	141818,18	1577272,7	70
S196	9090909,09	500	60090,91	1800000	1672727,3	187727,27	10
S197	13663636,36	450	120909,09	2559090,9	1481818,2	3972727,3	0
S198	1359090,91	50	2181,82	257272,73	31000	315454,55	0
S199	1709090,91	2300	3000	355909,09	231363,64	365909,09	55
S200	2113636364	550	14045,45	23272727	2259090,9	1,477E+09	0
