# Supplementary material for: Knowledge, Attitudes, and Hygienic Practices of Boiled Hypocotyls (Borassus aethiopum Mart) Vended in the Streets of Cotonou City and Its Outskirts, Benin
Source: Biomed Res Int. 2018 Aug 27;2018:4825435. doi: 10.1155/2018/4825435 (PMC6129848; doi:10.1155/2018/4825435)
Supplement: Supplementary Materials — The data used to support the findings of this study are included within the supplementary files. This section includes the survey sheet, the examination of survey cards, and the results of microbiological analyzes. [file 4825435.f1.zip › 4825435.f1/Survey Sheet.pdf]

SURVEY FORM: EVALUATION OF THE PRACTICE OF HYGIENE RULES FOR RONIER HYPOCOTYL TRANSFORMERS (*BORASSUS AETHIOPUM*)

Introduction

The hypocotyl of the *B.aethiopum* is very popular and is sold boiled in the streets of the city of Cotonou. This survey aims to know the processing conditions, the treatment of unsold products, the production chart, and to evaluate the hygiene practices of the processors during processing.

Sheet n°………

Date of Survey ……/……./………….

District …………………………………………………………

Borough number ………

City of …………………………………………

Last name and first names:…………………………………………………………

…………………………………………………………………………………………….

GPS coordinates found:..

A. IDENTIFICATION

**1. Sex**	M<br>F
**2. Age (years)**	a) < 15<br>b) 15 – 20<br>c) 20 – 25<br>d) 25 – 30<br>e) 30 – 35<br>f) 35 – 40<br>g) 40 – 45<br>h) 45 – 50<br>i) > 50
**3. Level of instruction**	a) Literate<br>b) Primary<br>c) Secondary<br>d) Others
**4. Kind of activities**	a) Producer of hypocotyl<br>b) Seller of hypocotyl<br>c) Producer and seller<br>d) Others

B. OVERVIEW OF THE CONDITIONS FOR PROCESSING OF BOILED *AGONTE*

5. Do you produce the tubers?

a) Yes

b) No

6. Where do you buy the tubers?

a) Market

b) In the field

7. Is it you who transforms?

a) Yes

b) No

8. Do you have any material reserved solely for cooking and for sale??

a) Yes

b) No

9. Where do you usually cook?

a) Outdoor

b) In the kitchen

Brick kitchen

Bread kitchen

10. Do you use detergent products to wash your utensils?

a) Yes

b) No

11. Do you know that hand washing should be done before and after all your tasks during the preparation of the tubers??

a) Yes

b) No

C. CRITICAL POINTS OF HYGIENE

12. Know the rules of hygiene?

a) Yes

b) No

13. What water do you use often for cooking?

a) Well water

b) Tap water

c) Other (specify)

14. What treatment is water treated when it is taken off the tap??

a) Add bleach

b) Add alum

c) Heating up to boiling

d) None

15. How often do you clean your utensils?

a) Often

b) Rarely

16. Do you know that microorganisms exist?

a) Yes

b) No

17. Do you know that they can cause diseases?

a) Yes

b) No

18. Do you have any idea of the routes of transmission of these microorganisms?

a) Unsuitable water

b) Dirty hands

c) Dirty utensils

d) Unknown

19. Hygienic conditions of the sales site

a) Clean

b) Dirty

20. Hygiene of the saleswoman

a) Lack of hygiene

b) Clean

21. Presence of garbage or dumps on the site of sale or cooking

a) Yes

b) No

22. Type of seller

a) Stationary

b) Ambulant

23. Packaging hot tubers

a) In coolers

b) In bulk and covered by a plastic bag

c) Loose and covered by a perforated canvas

d) Loose and covered by a non-perforated canvas

e) In recycled jute bag

f) Others

24. Treatment of unsolds and conservation

....

....

....

....

....

....

....

....

....

....

....

....

....

D. PRODUCTION DIAGRAM OF BOILED *AGONTE*
